# Synaptic coupling of inner ear sensory cells is controlled by brevican-based extracellular matrix baskets resembling perineuronal nets

**DOI:** 10.1186/s12915-018-0566-8

**Published:** 2018-09-26

**Authors:** Mandy Sonntag, Maren Blosa, Sophie Schmidt, Katja Reimann, Kerstin Blum, Tobias Eckrich, Gudrun Seeger, Dietmar Hecker, Bernhard Schick, Thomas Arendt, Jutta Engel, Markus Morawski

**Affiliations:** 1 Paul-Flechsig-Institute of Brain Research, Medical Faculty, University of Leipzig, Leipzig, Germany; 2 Department of Biophysics, Center for Integrative Physiology and Molecular Medicine (CIPMM), School of Medicine, Saarland University, Homburg, Germany; 3 Department of Otorhinolaryngology, School of Medicine, Saarland University, Homburg, Germany

**Keywords:** Brevican, Cochlea, Inner hair cell, Ribbon synapse, Extracellular matrix, Perineuronal net

## Abstract

**Background:**

Perineuronal nets (PNNs) are specialized aggregations of extracellular matrix (ECM) molecules surrounding specific neurons in the central nervous system (CNS). PNNs are supposed to control synaptic transmission and are frequently associated with neurons firing at high rates, including principal neurons of auditory brainstem nuclei. The origin of high-frequency activity of auditory brainstem neurons is the indefatigable sound-driven transmitter release of inner hair cells (IHCs) in the cochlea.

**Results:**

Here, we show that synaptic poles of IHCs are ensheathed by basket-like ECM complexes formed by the same molecules that constitute PNNs of neurons in the CNS, including brevican, aggreccan, neurocan, hyaluronan, and proteoglycan link proteins 1 and 4 and tenascin-R. Genetic deletion of brevican, one of the main components, resulted in a massive degradation of ECM baskets at IHCs, a significant impairment in spatial coupling of pre- and postsynaptic elements and mild impairment of hearing.

**Conclusions:**

These ECM baskets potentially contribute to control of synaptic transmission at IHCs and might be functionally related to PNNs of neurons in the CNS.

**Electronic supplementary material:**

The online version of this article (10.1186/s12915-018-0566-8) contains supplementary material, which is available to authorized users.

## Background

In addition to neurons and glia, the extracellular matrix (ECM) forms a fundamental, non-cellular component of the nervous system. A specialized form of the ECM are the perineuronal nets (PNNs), a pericellular cover that tightly enwraps somata, proximal dendrites, and axon initial segments of specific neurons thereby leaving meshes occupied by synaptic terminals. PNNs are composed of multiple extracellular molecules that interact with each other [1–5]. Among those are the proteoglycans aggrecan, neurocan, and brevican, which carry negatively charged chondroitin-sulfated side chains and determine the anionic nature of the PNN [1, 3]. The proteoglycans bind to hyaluronan that is anchored in the neuronal membrane and forms the scaffold of the PNNs [4]. Link proteins stabilize the connection between proteoglycans and hyaluronan, and finally, tenascin-R crosslinks proteoglycan-hyaluronan complexes [3, 6].

PNNs are associated with numerous functions, including neuroprotection [7–11], stabilization of synaptic contacts [12, 13], modulation of synaptic plasticity, and learning and memory processes [5, 12, 14, 15]. In addition, PNNs are assumed to contribute to high-frequency neuronal activity and synaptic transmission [16, 17]. They typically surround fast-spiking, parvalbumin-positive interneurons within the cortex [9, 16, 18]. PNNs are also densely expressed within all auditory brainstem nuclei, thereby surrounding various neuron types such as glutamatergic bushy cells in the cochlear nucleus or glycinergic principal neurons in the medial nucleus of the trapezoid body (MNTB) (for review see [19]). Depending on the acoustic input, these neuron types are able to operate at very high discharge rates. The origin of this high-rate activity is found in the cochlea, where inner hair cells (IHCs) transduce mechanical stimuli into receptor potentials, followed by faithful and indefatigable transmitter release at their ribbon synapses. This is transformed into action potentials by spiral ganglion neurons and further conveyed to the auditory brainstem [20, 21]. The question arises whether PNNs or a similar form of ECM also appear at IHCs despite their peripheral location and their epithelial origin.

Though many regions within the cochlea such as basilar membrane, spiral limbus, spiral ligament, and stria vascularis are highly enriched in ECM, including collagen, fibronectin, and various unspecified proteoglycans [22–24], the IHC-associated ECM has not yet been examined in detail. An earlier study by Santi and colleagues [25] reported an ECM coat made up of glycoconjugates at the endolymphatic surface of hair cells but specific molecules have not been identified. Thus, it remains largely elusive whether PNN-specific chondroitin-sulfated proteoglycans (CSPGs) of the lectican family (aggrecan, brevican, neurocan, and versican) are expressed within the cochlea at all. In addition, two of these lecticans, brevican, and neurocan are even suggested to be solely expressed in the central nervous system (CNS) [26–30].

Here, we aim to clarify whether cochlear hair cells exhibit an ECM coating comparable to PNNs of neurons within the CNS. The present data demonstrate that the main PNN molecules are expressed in the cochlea, including aggrecan, brevican, neurocan, the hyaluronan, and proteoglycan link proteins (HAPLN) 1 and 4, and tenascin-R. These ECM molecules were tightly associated with synaptic contacts, thereby forming basket-shaped complexes surrounding the base of IHCs. Among the proteoglycans brevican was found to be the major molecule constituting this ECM basket. Brevican-deficient mice displayed a remarkable degradation of the ECM basket surrounding IHCs accompanied by an increase in the number of dislocated postsynaptic densities relative to presynaptic calcium channels.

In conclusion, the present data demonstrate that the main PNN-constituting molecules are expressed in the cochlea, thereby forming brevican-based ECM baskets that enclose the base of IHCs and their synaptic contacts. Brevican potentially contributes to spatial coupling of pre- and postsynaptic elements and thus may support fast and temporally precise synaptic transmission in the cochlea.

## Results

### Distribution of PNN-constituting proteoglycans and link proteins in the cochlea

Given the sparse information on the expression of the CSPGs brevican, aggrecan, and neurocan, as well as HAPLN1, HAPLN4, and tenascin-R in the cochlea, the initial aim of the present study was to test whether these typical constituents of PNNs can be identified within cochlear tissue and whether these components are associated with cochlear sensory cells. Immunolabeling of those proteins was performed on cochlear cryosections prepared from 3 C57BL/6N mice at the age of P27–P30, complemented by Western blot analyses on 30 isolated cochleae of 15 C57BL/6N mice (P35).

**Fig. 1:**
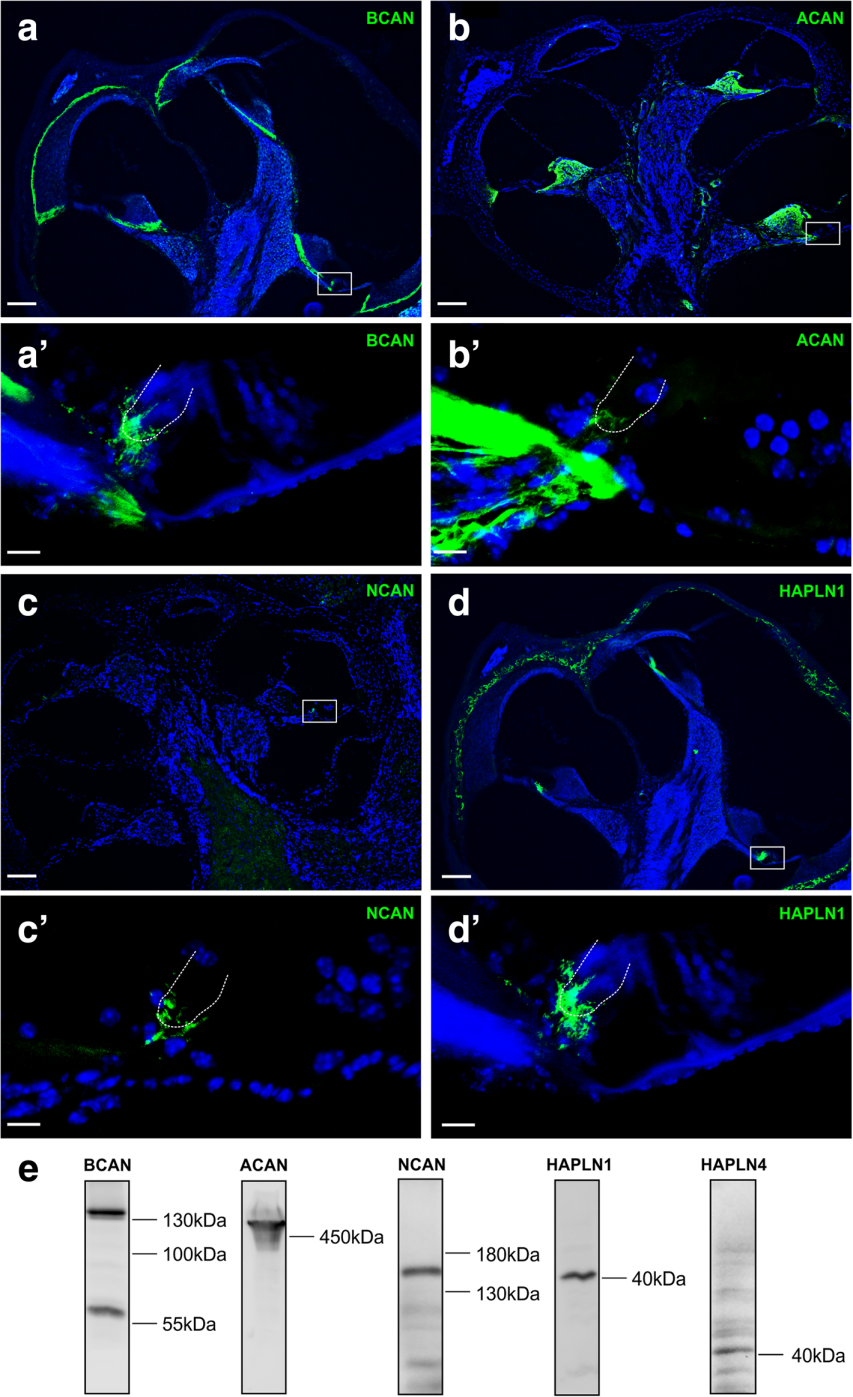
Immunohistochemical localization of chondroitin-sulfated proteoglycans (CSPGs) of the lectican family and hyaluronan and proteoglycan link protein 1 (HAPLN1) in cross-sections of the mouse cochlea. **a** and **a′** The immunoreaction of the CSPG brevican (BCAN) was detected in a layer between the spiral ligament and the temporal bone and in the osseous spiral lamina between the spiral limbus and the auditory nerve. BCAN also appeared prominently at inner hair cells (IHC) (**a′**, magnification of white box in **a**). **b** and **b′** Aggrecan (ACAN) labeling yields a strong immunosignal in the spiral limbus and partly in the spiral ligament**.** The magnification of the basilar membrane (**b**′, white box in **b**) reveals a weak staining of ACAN also at IHCs. **c** and **c′** The immunohistochemical identification of neurocan (NCAN) resulted in a positive signal only at IHCs (**c**′, magnification of white box in **c**) while the rest of the cochlea seemed to be spared of NCAN. **d** and **d′** HAPLN1 was detected in cochlear tissue in both the temporal bone and at IHCs (**d**′, magnification of white box in **d**). **e** Western blot analysis confirmed the presence of BCAN (~ 145 kDa full-length protein and 55 kDa fragment), ACAN (~ 450 kDa), NCAN (~ 150 kDa), HAPLN1 (~ 40 kDa), and HAPLN4 (~ 40–42 kDa) in the cochlear tissue of the mouse. Note that in **a** and **a**′, BCAN was labeled by a Cy3 secondary antibody. For better comparison the red color was switched to green. **a**–**d** and **a′**–**d**′ Maximum intensity projections of confocal stacks of cochlear cross-sections. **a**–**d**, scale 100 μm. **a**′–**d**′, scale 10 μm

All of the tested CSPGs as well as HAPLN1 could be identified in cross-section of the cochlea (Fig. 1), even brevican (Fig. 1a) and neurocan (Fig. 1c), which have been presumed to be specific for the brain [26–29].

Brevican appeared as a thin layer both (i) underneath the spiral limbus within the osseous spiral lamina and (ii) between the spiral ligament and the bony otic capsule (Fig. 1a). These brevican-positive layers are likely to reflect basement membranes, which are thin layers between cells and connective tissues and were shown to be enriched in CSPGs [24]. Surprisingly, there was an additional strong brevican immunosignal at IHCs (Fig. 1a′). Aggrecan was intensively labeled in the spiral limbus and in the lower part of the spiral ligament, corresponding to the region of type IV fibrocytes (Fig. 1b). We further detected a rather weak yet specific aggrecan signal at IHCs (Fig. 1b′). A similar delicate but clearly positive immunoreactivity at IHCs was observed for the CSPG neurocan (Fig. 1c′) which obviously did not appear anywhere else within the cochlea (Fig. 1c). Among the tested proteoglycans, the immunosignal of brevican was the strongest. HAPLN1 was identified within the otic capsule and also revealed a strikingly strong immunosignal at IHCs (Fig. 1d, d′). Double-staining with an anti-calbindin antibody which labels cochlear hair cells confirms that the immunohistochemical signal of HAPLN1 is specifically surrounding the IHC (Additional file 1: Figure S1).

Western blot analyses confirmed the presence of brevican (~ 145 kDa full-length protein and ~ 55 kDa fragment), aggrecan (~ 450 kDa), neurocan (~ 150 kDa), HAPLN1 (~ 40 kDa), and HAPLN4 (~ 40–42 kDa) (Fig. 1e) in the mouse cochlea. HAPLN4 could be immunohistochemically identified in cochlear whole-mounts (see below) but not in cochlear cross-sections, probably due to epitope alteration caused by the decalcification process. Tenascin-R immunohistochemistry in cochlear cross-sections and Western blot analyses did not yield positive results, but this ECM molecule could successfully be identified in whole-mounts (see below).

In conclusion, we found that the main PN components are present in the auditory periphery. Despite of the fact that brevican, aggrecan, neurocan, and HAPLN1 exhibit an individual distribution within the cochlea, they conjointly appeared at IHCs, potentially pointing to the presence of an extracellular, PNN-like structure surrounding the sensory cells of the cochlea.

### CSPGs, HAPLN1, and HAPLN4 form basket-like ECM complexes around synapses at cochlear inner hair cells

**Fig. 2:**
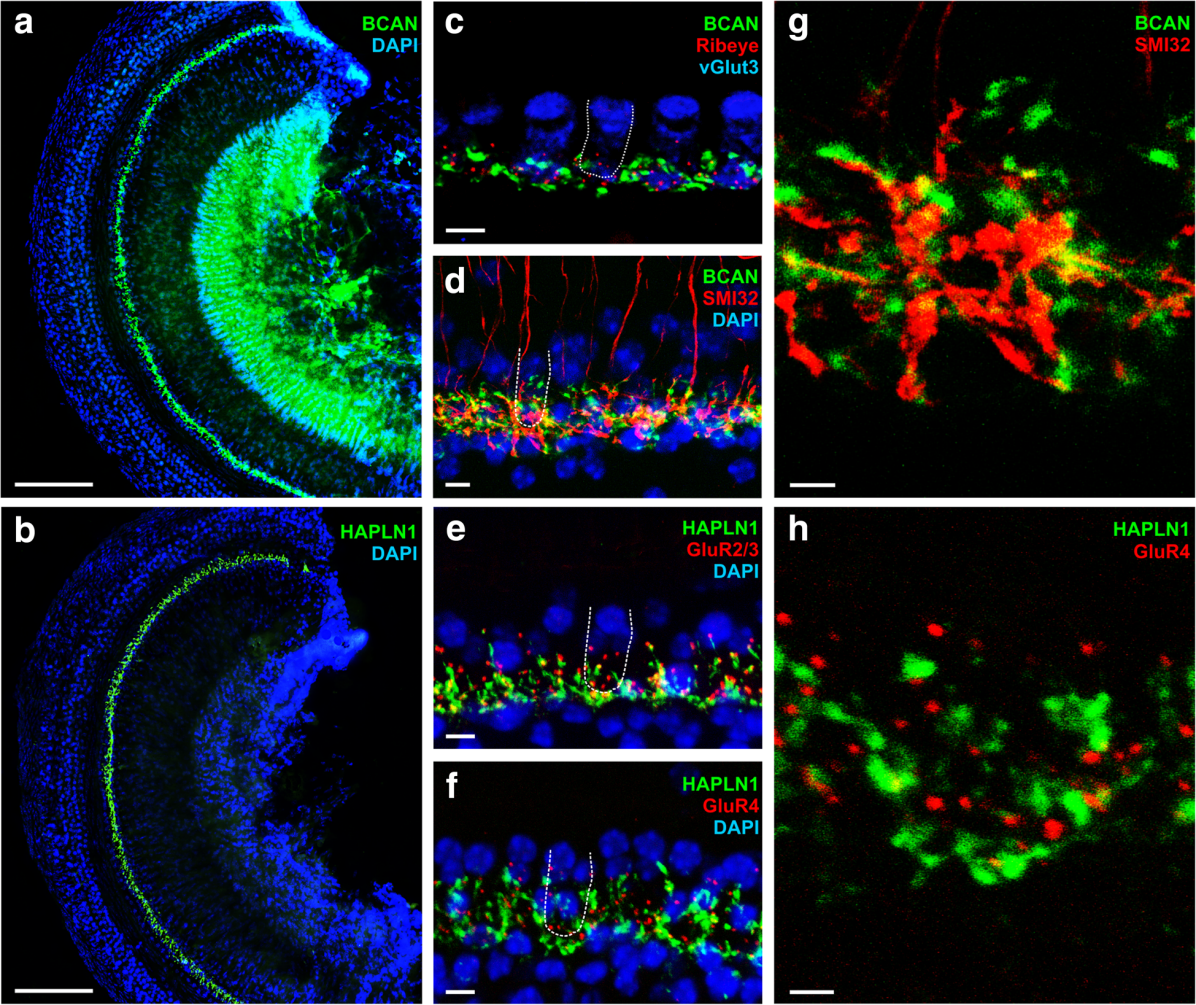
Localization of brevican and HAPLN1 in whole-mount preparations of the mouse cochlea. **a**, **b** Immunolabeling of brevican (BCAN, **a**) and HAPLN1 (**b**) revealed an extensive accumulation of these ECM molecules at IHCs. **c**–**f** Double-immunolabeling with the Ribeye marker CtBP2 (red, **c**), the neurofilament marker SMI32 (red, **d**), and antibodies against GluR2/3 (red, **e**), and GluR4 (red, **f**) showed that brevican (green, **c**, **d**) and HAPLN1 (green, **e**, **f**) were expressed at the base of the IHCs in the region where the pre- and postsynaptic elements of IHC synapses are located. **g**, **h** Magnifications of indicated IHCs in **d** and **f** revealed a basket-like formation of ECM at IHCs, with brevican (green, **g**) tightly enclosing terminating fibers (SMI32, red, **g**) and HAPLN1 (green, **h**) forming gaps in which glutamate receptors are located (GluR4, red, **h**). **a-h** Maximum intensity projections of confocal stacks of whole-mount preparations from apical turns of organs of Corti. Nuclei are stained with DAPI (blue). In **c**, IHCs are marked with an anti-vGlut3 antibody (blue). In **d**–**f**, an exemplary IHC is indicated by the dashed outline. **a**, **b** Scale 100 μm. **c**–**f** Scale 5 μm. **g**, **h** Scale 2 μm

**Fig. 3:**
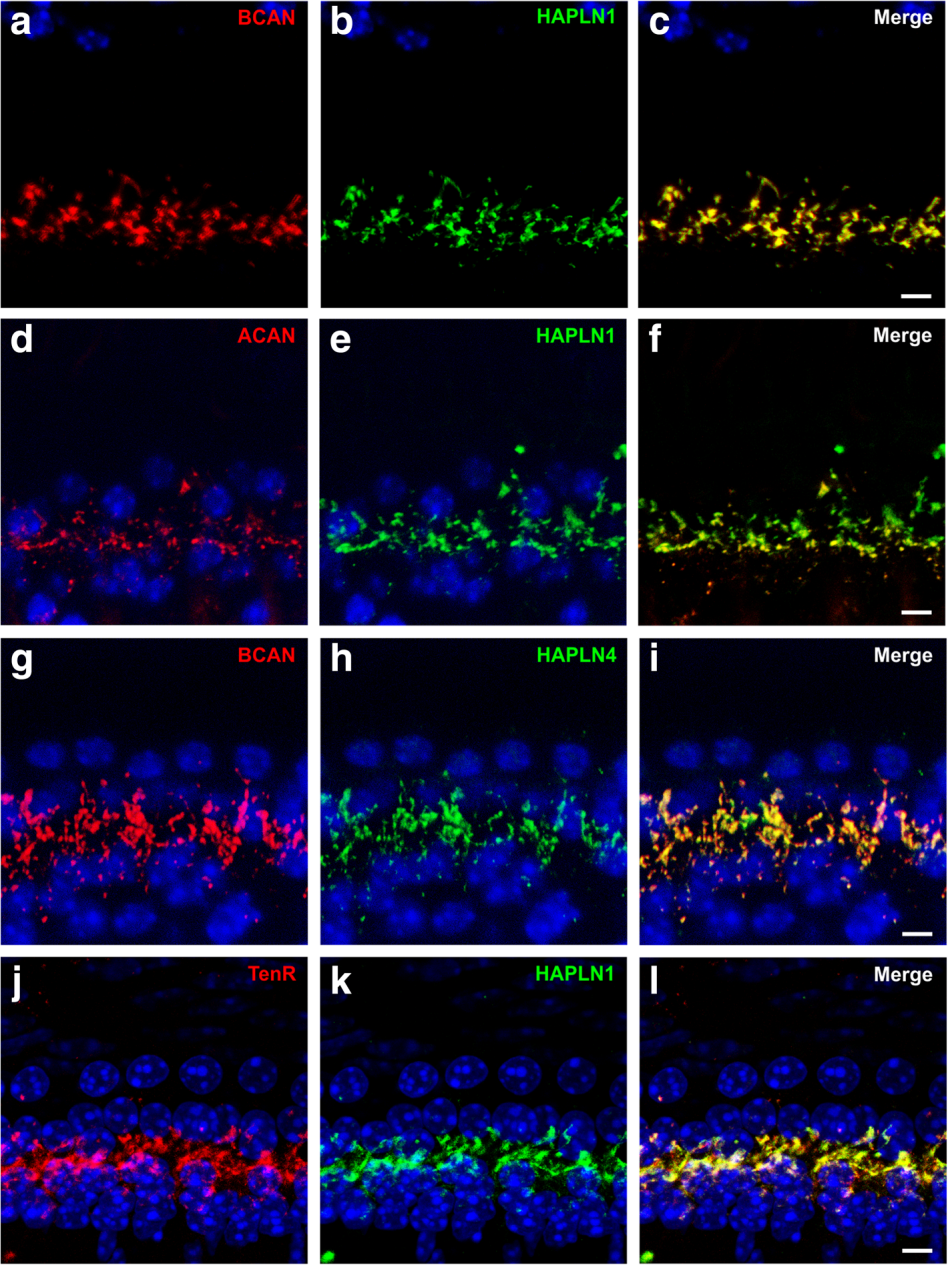
Spatial relationship of brevican, aggrecan, HAPLN1, HAPLN4, and tenascin-R in whole-mount preparations of the mouse cochlea. **a**–**c** The immunosignal of brevican (**a**, **c**, red) largely overlapped with that of HAPLN1 (**b**, **c**, green). **d**, **e** Double-staining of aggrecan (**d**, **f** red) and HAPLN1 (**e**, **f** green) also demonstrated a close spatial correlation between these ECM molecules. **g-i** Brevican immunoreactivity (**g**, **i** red) appeared to be almost completely colocalized with that of HAPLN4 (**h**, **i** green). **j**–**l** Tenascin-R (TenR) immunoreactivity (**j**, **l** red) yielded large overlap with HAPLN1 immunoreactivity (**k**, **l** green). **a**–**l** Maximum intensity projections of confocal stacks of stretches of 5–6 IHCs from apical cochlear turns with the nuclei stained with DAPI (blue), scales 5 μm

Next, we focused on the distribution and interaction of ECM molecules surrounding the cochlear hair cells. Whole-mount cochlear sections of young adult C57BL/6N mice (P26–P30, *n* = 15) were prepared, and ECM molecules were labeled using fluorescence immunohistochemistry. The labeling of brevican and HAPLN1 confirmed the prominent presence of these ECM molecules at IHCs along the entire cochlear spiral (Fig. 2a, b). We did not observe any differences in the distribution of brevican and HAPLN1 between apical and basal IHCs. The magnification of individual IHCs revealed that brevican and HAPLN1 exclusively embraced their basolateral poles covering the region where synaptic contacts were formed, demonstrated by immunohistochemical detection of pre- and postsynaptic structures, e.g., the ribbon synapses (Ribeye marker CtBP2; Fig. 2c), terminating fibers (neurofilament marker SMI32, Fig. 2d), and postsynaptic glutamate receptors (GluR2/3 and GluR4, Fig. 2e, f). Further magnification indicated that brevican and HAPLN1 enclose the hair cells in a basket-like manner, tightly enwrap terminating fibers (Fig. 2g), and form gaps in which synaptic elements are located (Fig. 2h). Similar distributions were found for aggrecan, neurocan, HAPLN4, and tenascin-R (for examples see Figs. 3 and 6c, g, i).

Immunohistochemical co-labeling revealed a strong spatial relationship between the CSPGs and link proteins (Fig. 3). Brevican was found to strongly overlap with HAPLN1 (Fig. 3a–c). Similar observations were made for aggrecan and HAPLN1 though aggrecan immunosignal was much weaker than HAPLN1 (Fig. 3d–f). Double-labeling of brevican and HAPLN4 (Fig. 3g–i) and tenascin-R and HAPLN1 (Fig. 3j–l) also revealed an almost complete co-localization of these ECM molecules. These data imply that the proteoglycans aggrecan and brevican form complex aggregates with HAPLN1, HAPLN4, and tenascin-R, resulting in basket-like envelopes surrounding the base of IHCs. The strong association with synaptic structures and the similarities in molecular composition suggest that these ECM baskets at the IHCs are structurally and functionally related to PNNs of neurons in the CNS.

### Brevican and HAPLN1 are also associated with ribbon synapses at outer hair cells

**Fig. 4:**
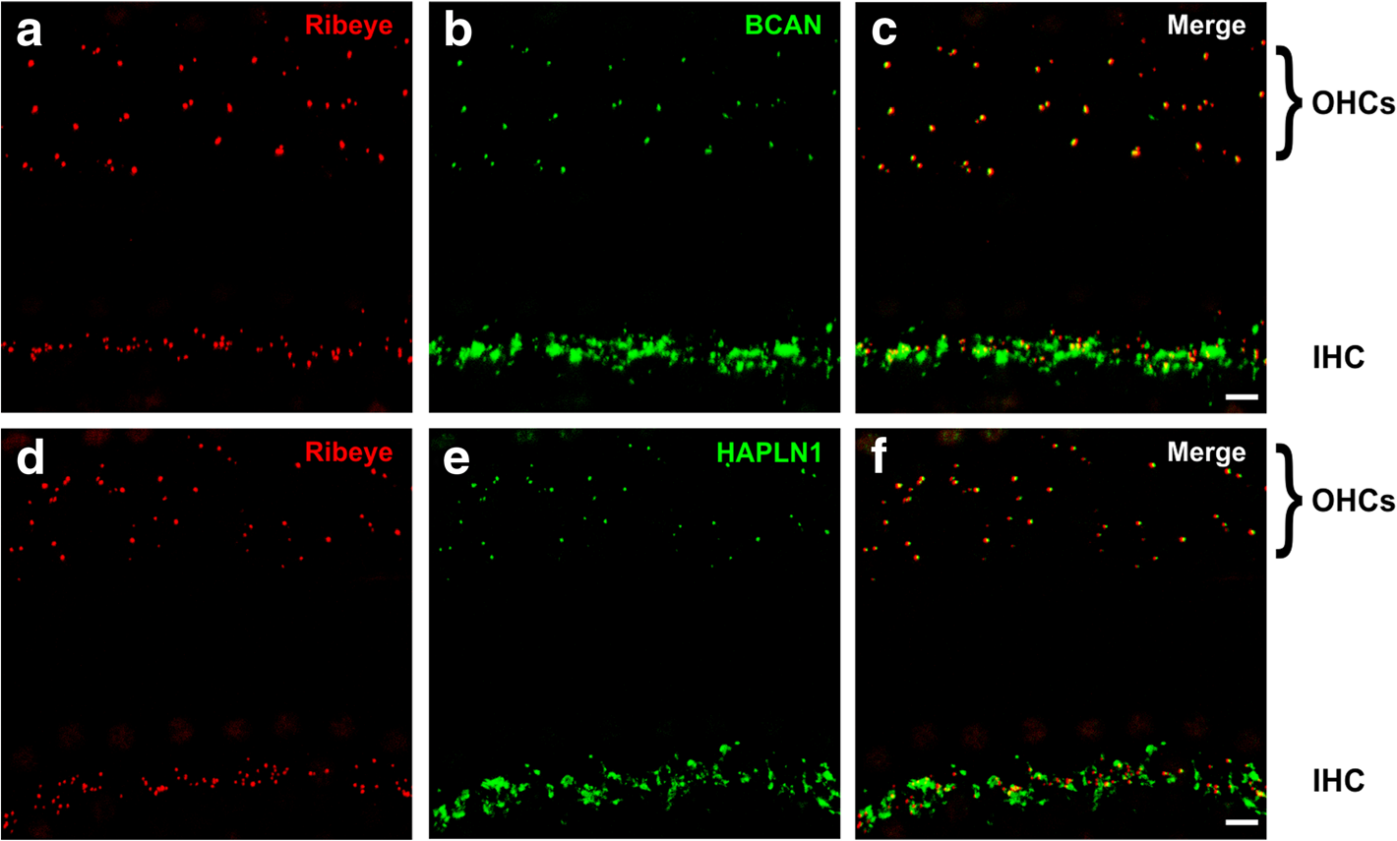
Distribution of brevican and HAPLN1 at OHCs in whole-mount preparations of the mouse cochlea. **a**–**c** Ribbon synapses in the three rows of OHCs and in the IHC row were labeled by the Ribeye marker CtBP2 (**a**, **c**, red). Besides its strong immunoreactivity in the IHC row, brevican occasionally also appeared in form of small punctae opposite to the ribbons of OHCs (**b**, **c**, green). **d**–**f** The labeling of HAPLN1 (**e**, **f**, green) also resulted in small immuno-positive punctae opposite to the ribbon synapses of OHCs (**d**, **f**, red). **a-f** Maximum intensity projections of confocal stacks of stretches of 5–6 IHCs from apical cochlear turns, scales 5 μm

Since outer hair cells (OHCs) also convey afferent information via ribbon synapses and in addition receive three to four efferent fibers, we questioned whether brevican, aggrecan, neurocan, HAPLN1, and HAPLN4 as well as tenascin-R also surround synapses at OHCs. In the majority of cochlear cross-sections (see Fig. 1) and of cochlear whole-mount preparations that were analyzed in the present study (Fig. 2a, b), we only occasionally observed immuno-positive spots at OHCs. Very rarely a larger number of OHCs within a single whole-mount section were associated with ECM (for example see Fig. 4), but this observation was not linked to the position of the OHCs within the organ of Corti. Further, only brevican (Fig. 4a–c) and HAPLN1 (Fig. 4d–f) could be detected at OHCs, whereas neither aggrecan, neurocan, HAPLN4, nor tenascin-R could be identified. The distribution of brevican and HAPLN1 at OHCs differed from that at IHCs. In comparison to the ECM basket enclosing the entire basal pole of IHCs, the labeling of brevican and HAPLN1 at OHCs only appeared as small punctae in close apposition to ribbon synapses (Fig. 4c, f).

In conclusion, these data demonstrate that the size and composition of ECM at OHCs differ from that of IHCs if present at all. At IHCs, the proteoglycans aggrecan, brevican, and neurocan as well as HAPLN1, HAPLN4, and tenascin-R form complex ECM baskets surrounding synapses at the entire basal part of the IHCs. At OHCs, only brevican and HAPLN1 seemed to be present and these molecules were found to be spatially restricted to individual ribbon synapses, resulting in a spot-like immunoreactivity.

### Lack of brevican leads to structural degradation of ECM baskets at IHCs

**Fig. 5:**
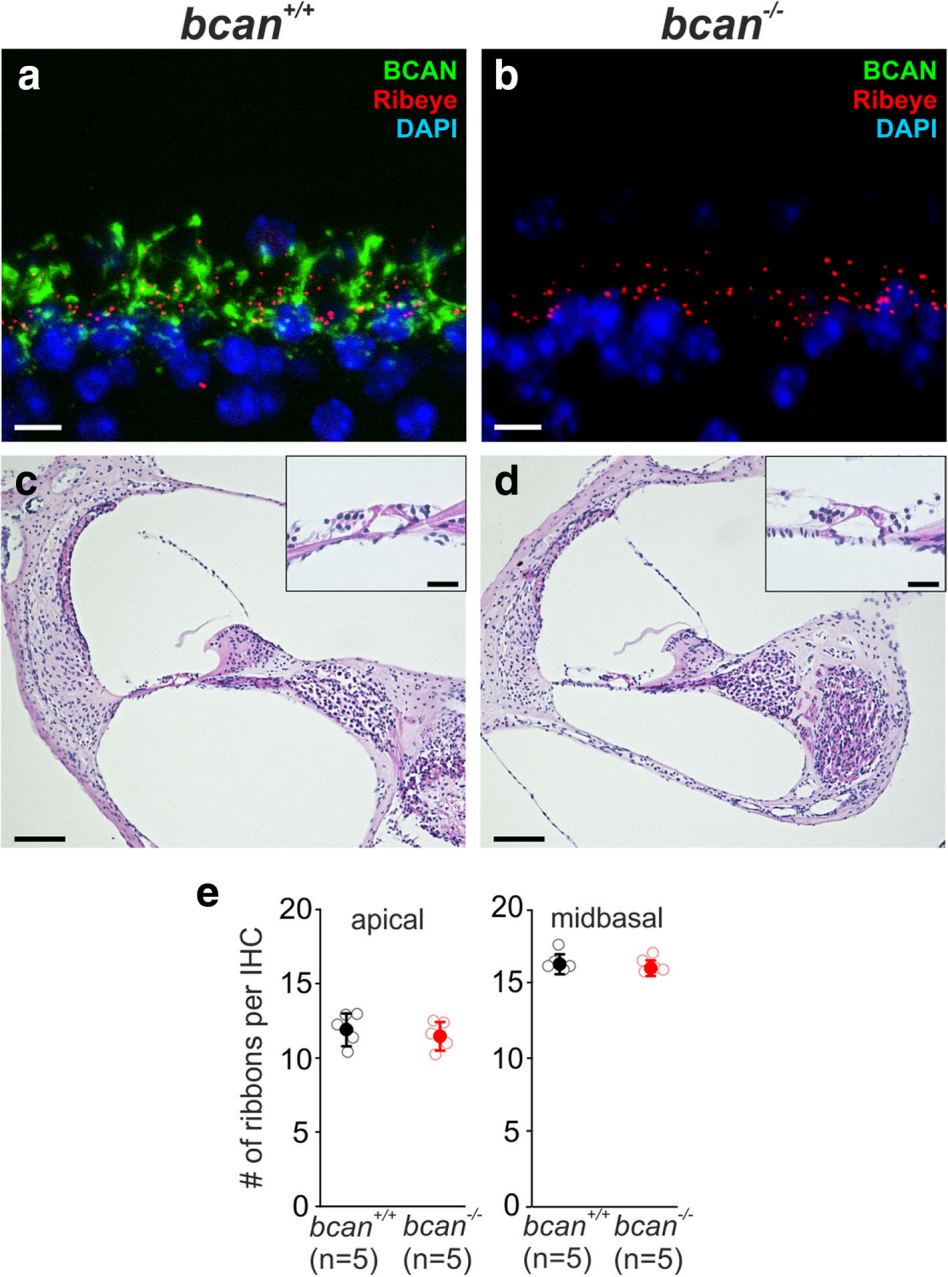
General cochlear morphology of brevican-deficient (*bcan*^*−/−*^) mice. **a**, **b** Immunohistochemical verification of the presence of brevican (BCAN) in wildtype (*bcan*^*+/+*^) mouse cochleae (**a**, green) and of its absence in *bcan*^*−/−*^ mouse cochleae (**b**, green). The ribbon synapses were labeled with the ribeye marker CtBP2 (**a**, **b**, red). Maximum intensity projections of confocal stacks of stretches of 5–6 IHCs of cochlear whole-mount preparations, scale 5 μm. **c**, **d** Hematoxylin-eosin staining of paraffin sections of *bcan*^*+/+*^ (**c**) and *bcan*^*−/−*^ mouse cochleae (**d**) did not reveal any obvious differences in the general morphology of cochlear tissue. A higher magnification of the basilar membrane is depicted in the insets. Scale 100 μm, scale inset 25 μm. **e** Quantification of the number of ribbon synapses (CtBP2-positive punctae) per IHC in apical and midbasal cochlear turns did not yield any genotype-specific differences (apical, *bcan*^*+/+*^: 11.8 ± 1.1, *n* = 5/38 whole-mounts/IHCs, *bcan*^*−/−*^: 11.4 ± 0.9, *n* = 5/40, *p* = 0.524, *t* test; midbasal, *bcan*^*+/+*^: 16.3 ± 0.7, *n* = 5/43, *bcan*^*−/−*^: 16.1 ± 0.5, *n* = 5/41, *p* = 0.62, *t* test). Data are presented as mean ± S.D

Among the CSPGs, brevican immunoreactivity seemed to be the strongest at IHCs. In order to estimate the significance of brevican surrounding IHCs, we analyzed a brevican-deficient mouse [17, 31]. Immunohistochemical staining of brevican confirmed the complete absence of this proteoglycan at IHCs in homozygous brevican knockout mice (*bcan*^−/−^) (Fig. 5a, b). At first, we focused on the general morphology of the cochlea in paraffin sections stained with hematoxylin-eosin. The cochleae of *bcan*^*−/−*^ mice revealed the typical gross features of wildtype cochleae without any obvious structural abnormalities (Fig. 5c, d). Also, the number of ribbon synapses was unaltered in *bcan*^*−/−*^ mice (Fig. 5e, apical, *bcan*^*+/+*^: 11.8 ± 1.1, *n* = 5/38 whole-mounts/IHCs, *bcan*^*−/−*^: 11.4 ± 0.9, *n* = 5/40, *p* = 0.524, *t* test; midbasal, *bcan*^*+/+*^: 16.3 ± 0.7, *n* = 5/43, *bcan*^*−/−*^: 16.1 ± 0.5, *n* = 5/41, *p* = 0.62, *t* test).

**Fig. 6:**
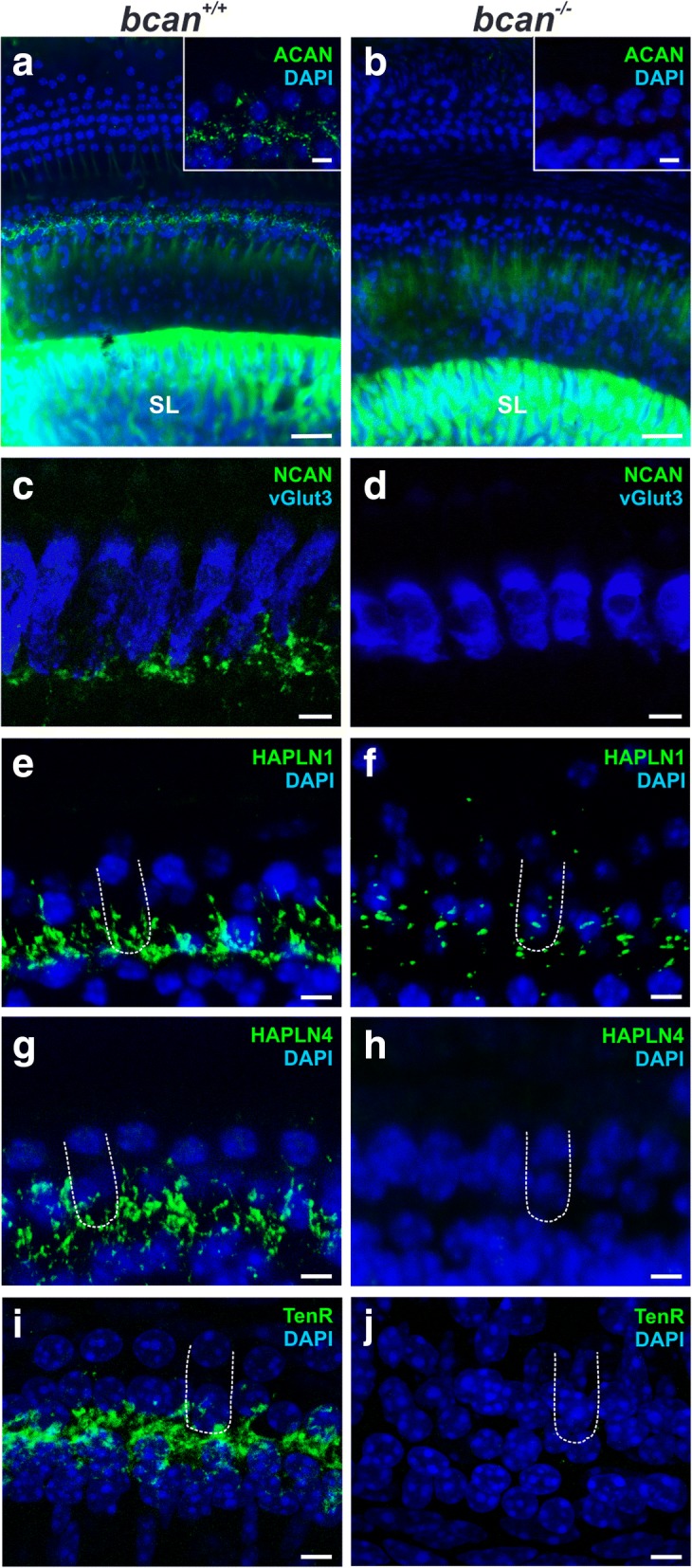
Distribution of CSPGs, HAPLN1, HAPLN4 and tenascin-R in cochlear whole-mount preparations of *bcan*^*−/−*^ mice. **a**, **b** Aggrecan labeling (ACAN, green) yielded a strong immunosignal in the spiral limbus (SL) in both *bcan*^*+/+*^ (**a**) and *bcan*^*−/−*^ mice (**b**). At IHCs, aggrecan was only detectable in *bcan*^*+/+*^ but not in *bcan*^*−/−*^ organs of Corti (magnification of 4–5 IHCs in the insets). **c**, **d** Neurocan (green) could be visualized at the base of IHCs in wildtype mice (**c**) but was absent at IHCs of *bcan*^*−/−*^ mice (**d**). **e**, **f** The immunoreactivity of HAPLN1 (green) is strongly reduced at IHCs in brevican-deficient mice (**f**) compared to wildtype mice (**e**). **g**, **h** The positive immunosignal of HAPLN4 (green) at the base of IHCs in wildtype mice (**g**) was absent in *bcan*^*−/−*^ mice (**h**). **i**, **j** The immunosignal of tenascin-R (green) at the base of IHCs in wildtype mice (**i**) was absent in *bcan*^*−/−*^ mice (**j**). Maximum intensity projections of confocal stacks of stretches of 30–40 IHCs (**a**, **b**) or 6–7 IHCs (**c**–**j**) of cochlea whole-mount preparations with nuclei stained with DAPI (**a**, **b**, **e**–**j**, blue) or with IHCs labeled by an anti-vGlut3 antibody (**c**, **d**, blue). Exemplary IHCs are indicated by the dashed outline. **a**, **b** scale 25 μm, insets 5 μm, **c-j** scale 5 μm

Next, we analyzed alterations in the molecular structure of ECM baskets at IHCs in *bcan*^*−/−*^ mice. The CSPGs aggrecan and neurocan as wells as HAPLN1, HAPLN4, and tenascin-R were labeled in cochlear whole-mount preparations of *bcan*^*+/+*^ and *bcan*^*−/−*^ mice, using fluorescence immunohistochemistry. The analysis yielded profound changes in the ECM composition at IHCs in *bcan*^*−/−*^ mice (Fig. 6). While aggrecan was strongly expressed in the spiral limbus of both *bcan*^*+/+*^ and *bcan*^*−/−*^ mouse cochleae (Fig. 6a, b), the weak aggrecan immunosignal found at wildtype IHCs was obviously absent from IHCs of *bcan*^*−/−*^ mice (Fig. 6a, b, insets). Similar observations were made for neurocan, which was not detectable at IHCs of *bcan*^*−/−*^ mice (Fig. 6c, d). Though HAPLN1 was still present at IHCs in *bcan*^*−/−*^ mice, the direct comparison to wildtype IHCs demonstrated a strong reduction of this protein in knockout animals (Fig. 6e, f). HAPLN4 did not yield any positive immunosignal at IHCs in *bcan*^*−/−*^ mice (Fig. 6g, h), and finally, also tenascin-R seemed to be virtually absent at IHCs in *bcan*^*−/−*^ mice (Fig. 6i, j).

In conclusion, the lack of brevican resulted in the loss of IHC-associated ECM. Besides brevican, the other two proteoglycans aggrecan and neurocan as well as HAPLN4 and tenascin-R were absent at IHCs in *bcan*^*−/−*^ mice. From the tested ECM components, only HAPLN1 was still detectable at IHCs in *bcan*^*−/−*^ mice, though its immunoreactivity was strongly reduced resulting in a spot-like pattern of this protein. Thus, brevican is an essential determinant of the structure and molecular composition of ECM baskets at IHCs.

### Calcium channel currents are not affected in IHCs of bcan^−/−^ mice

Due to the extensive changes in the ECM structure at IHCs in *bcan*^*−/−*^ mice, we hypothesized that the function of hair cells was affected in the mutant mice. This idea is supported by a recent study performing in vivo electrophysiological recordings in the MNTB of *bcan*^*−/−*^ mice demonstrating a significant decrease in sound-evoked firing rates. This change did not originate in the MNTB itself and may point to a disruption of hair cell function [17].

**Fig. 7:**
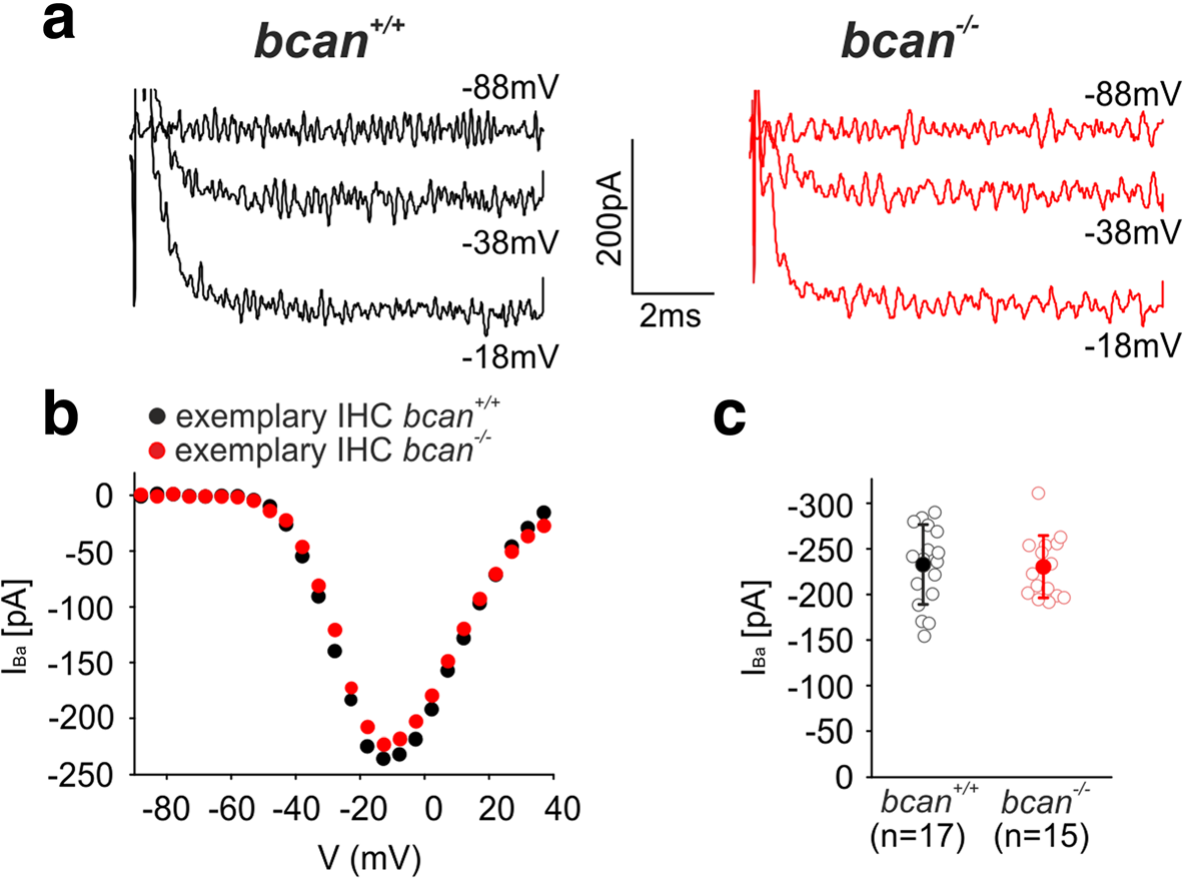
Ba^2+^ currents recorded in IHCs of *bcan*^*−/−*^ mice. **a** Exemplary current traces of an apical turn *bcan*^*+/+*^ IHC (black, left, P20) and an apical turn *bcan*^*−/−*^ IHC (red, right, P21) in response to 8 ms step depolarizations to the voltages indicated. **b** Corresponding *I-V*-curves of the exemplary cells depicted in **a** (*bcan*^*+/+*^ IHC: black; *bcan*^*−/−*^ IHC: red). **c** Average maximum Ba^2+^ currents did not point to any disruption of the function of presynaptic calcium channels in IHCs of *bcan*^*−/−*^ mice (*bcan*^*+/+*^: − 232 ± 43.6 pA, *n* = 17/2 IHCs/mice, *bcan*^*−/−*^: − 229.5 ± 34 pA, *n* = 15/2, *p* = 0.861, *t* test). Data are presented as mean ± S.D

To test for changes in IHC function, whole-cell Ca^2+^ channel currents with Ba^2+^ as charge carrier were recorded in apical turn IHCs of 3-week-old *bcan*^*+/+*^ (*n* = 2) and *bcan*^*−/−*^ mice (*n* = 2, Fig. 7). Exemplary current traces in response to 8-ms step depolarizations to the voltages indicated (Fig. 7a, left, black: *bcan*^*+/+*^; right, red: *bcan*^*−/−*^ mice) and corresponding *I-V* curves (Fig. 7b, *bcan*^*+/+*^: black, *bcan*^*−/−*^: red) did not indicate any genotype-specific changes in IHC Ba^2+^ currents. Also, average maximum Ba^2+^ currents were not altered in *bcan*^*−/−*^ mice (Fig. 7c, *bcan*^*+/+*^: − 232 ± 43.6 pA, *n* = 17; *bcan*^*−/−*^: − 229.5 ± 34 pA, *n* = 15, *p* = 0.861, *t* test). Gating parameters of the Ca^2+^ channels such as the voltage of the half-maximum activation, *V*_*h*_, and the slope of activation, *k*, were extracted from fits to the *I-V* curves (see the “Methods” section). There was no difference in *V*_*h*_between *bcan*^*+/+*^ (− 28.8 ± 2 mV, *n* = 17) and *bcan*^*−/−*^ mice (− 29.1 ± 2.6 mV, *n* = 15, *p* = 0.59, *t* test). Likewise, the slope *k* was not different between genotypes (*bcan*^*+/+*^: 10.44 ± 0.57 mV, *n* = 17; *bcan*^*−/−*^: 10.46 ± 0.6 mV, *n* = 15; *p* = 0.94, *t* test). To summarize, brevican does not affect the function of presynaptic calcium channels.

### Hearing thresholds and auditory processing are slightly impaired in bcan^−/−^ mice

**Fig. 8:**
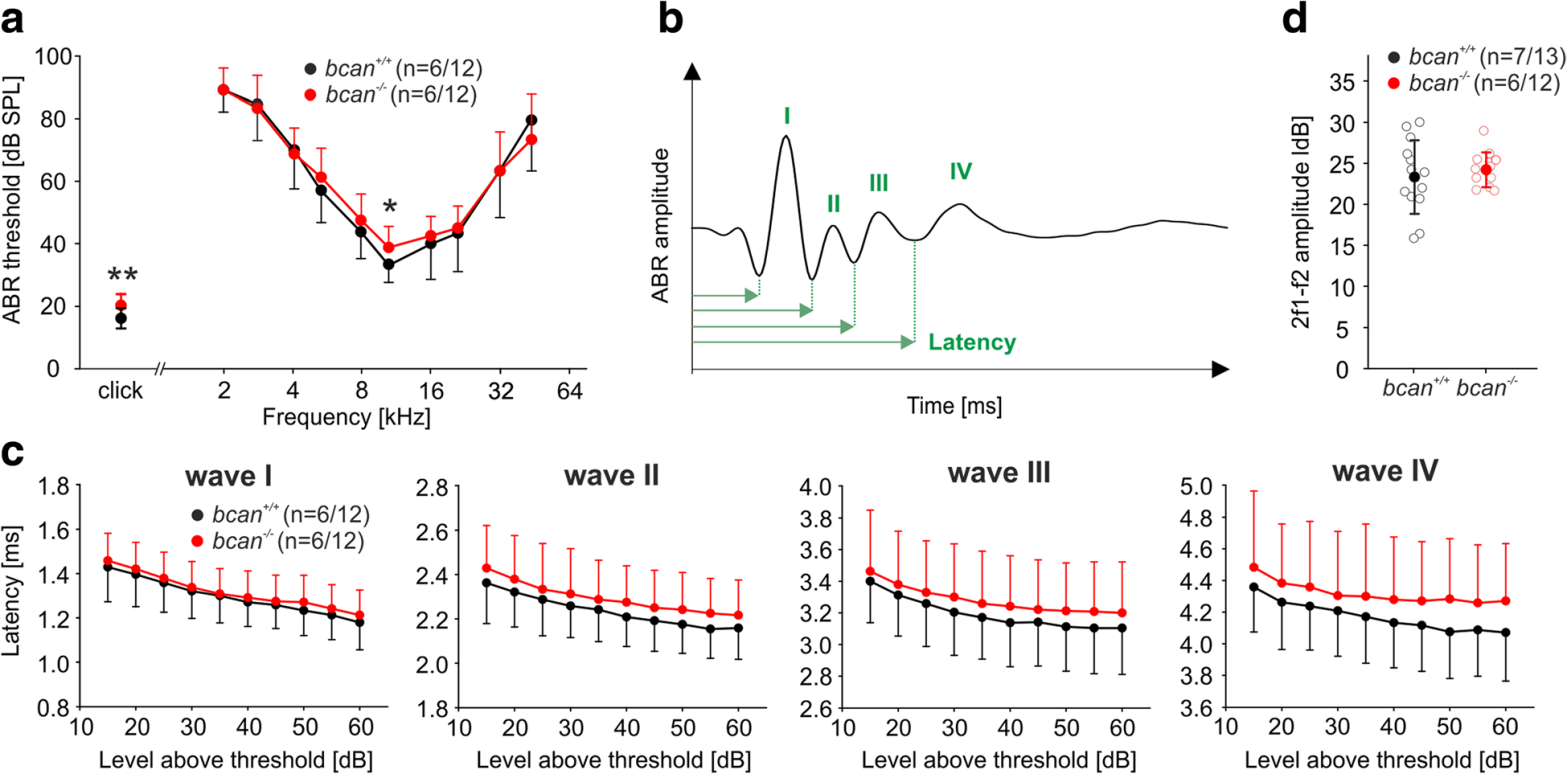
Hearing function assessed by auditory brainstem responses (ABR) and distortion product otoacoustic emissions (DPOAE) in *bcan*^*−/−*^ mice. **a** ABR thresholds of *bcan*^*−/−*^ mice (red) were elevated compared with wildtype mice (black) in response to both click stimuli (*bcan*^*+/+*^: 16.7 ± 3.3 dB SPL, *n* = 6/12 animals/ears; *bcan*^*−/−*^: 20.0 ± 3.6 dB SPL, *n* = 6/12 animals/ears, *p* < 0.01, *t* test) and pure tones, which covered the frequencies of best hearing of mice (at 11.3 kHz, *bcan*^*+/+*^: 33.3 ± 6.8 dB SPL, *n* = 6/12 animals/ears; *bcan*^*−/−*^: 38.8 ± 6.8 dB SPL, *n* = 6/12 animals/ears, *p* < 0.05, *t* test). **b** Sketch illustrating a click ABR waveform with waves I–IV indicated at the positive peaks, respectively, and the definition of latency as the time point of the negative (leading) peak of the individual wave. **c** Growth functions of the latencies of waves I to IV (mean ± S.D.) show consistently larger mean latencies for all four waves and all stimulus levels (except latency of wave I at 35 dB above threshold) in *bcan*^*−/−*^ mice (red) compared to wildtype mice (black; *n* = 6/12, animals/ears each genotype). For clarity, the S.D. is plotted in one direction only (+ S.D. or − S.D.). **d** DPOAE maximum amplitudes averaged over 10–18 kHz did not differ between genotypes (*bcan*^*+/+*^_,_ black: 23.3 ± 4.5 dB, *n* = 7/13 animals/ears; *bcan*^*−/−*^*,* red: 24.2 ± 2.1 dB, *n* = 6/12 animals/ears, *p* = 0.537, *t* test). * *p* < 0.05, ** *p* < 0.01. Data are presented as mean ± S.D

Next, we analyzed hearing performance of *bcan*^*−/−*^ mice. Click auditory brainstem response (ABR) thresholds were slightly but significantly increased in knockout mice (*bcan*^*+/+*^: 16.7 ± 3.3 dB sound pressure level (SPL), *n* = 6/12 animals/ears; *bcan*^*−/−*^: 20.0 ± 3.6 dB SPL, *n* = 6/12 animals/ears, *p* < 0.01, *t* test, Fig. 8a). Frequency-specific ABR thresholds acquired between 2 and 45 kHz (Additional file 2: Figure S2) yielded consistently elevated thresholds in the range between 5.6 and 22.6 kHz in *bcan*^*−/−*^ mice, covering the best hearing range of mice. The difference was the largest at 11.3 kHz (*bcan*^*+/+*^: 33.3 ± 6.8 dB SPL, *n* = 6/12 animals/ears; *bcan*^*−/−*^: 38.8 ± 6.8 dB SPL, *n* = 6/12 animals/ears, *p* < 0.05, *t* test, Fig. 8a).

Analysis of the latencies of individual ABR waves revealed timing differences between *bcan*^*+/+*^ and *bcan*^*−/−*^ mice (Fig. 8c). Latencies of ABR waves I to IV elicited by a click stimulus delivered at *t* = 0 were defined as the time point of the negative peak of the respective wave (Fig. 8b). Interestingly, growth functions (Fig. 8c, Additional file 3: Figure S3) revealed that latencies of wave I to IV tended to be prolonged in *bcan*^*−/−*^ mice for each stimulus level with the difference between genotypes becoming larger with increasing wave number. Due to the high variability between mice, these differences did not reach statistically significant levels, except the *y*-axis intercept of wave II latencies in a correlation analysis (*p* = 0.026). The results indicate that deficiency of brevican leads to mild impairment of central auditory signal processing.

We further tested OHC function by measuring distortion product otoacoustic emissions (DPOAEs), a measure of OHC electromotility (Fig. 8d). The 2f1-f2 DPOAE maximum amplitudes for f2 were averaged over 17 frequencies between 10 and 18 kHz and did not reveal any genotype-specific differences (*bcan*^*+/+*^: 23.3 ± 4.5 dB, *n* = 7/13 animals/ears; *bcan*^*−/−*^: 24.2 ± 2.1 dB, *n* = 6/12 animals/ears, *p* = 0.537, *t* test, Fig. 8d), indicating that OHC function was not impaired in absence of brevican.

### Spatial coupling of presynaptic Ca_v_1.3 and postsynaptic densities is reduced in brevican-deficient mice

**Fig. 9:**
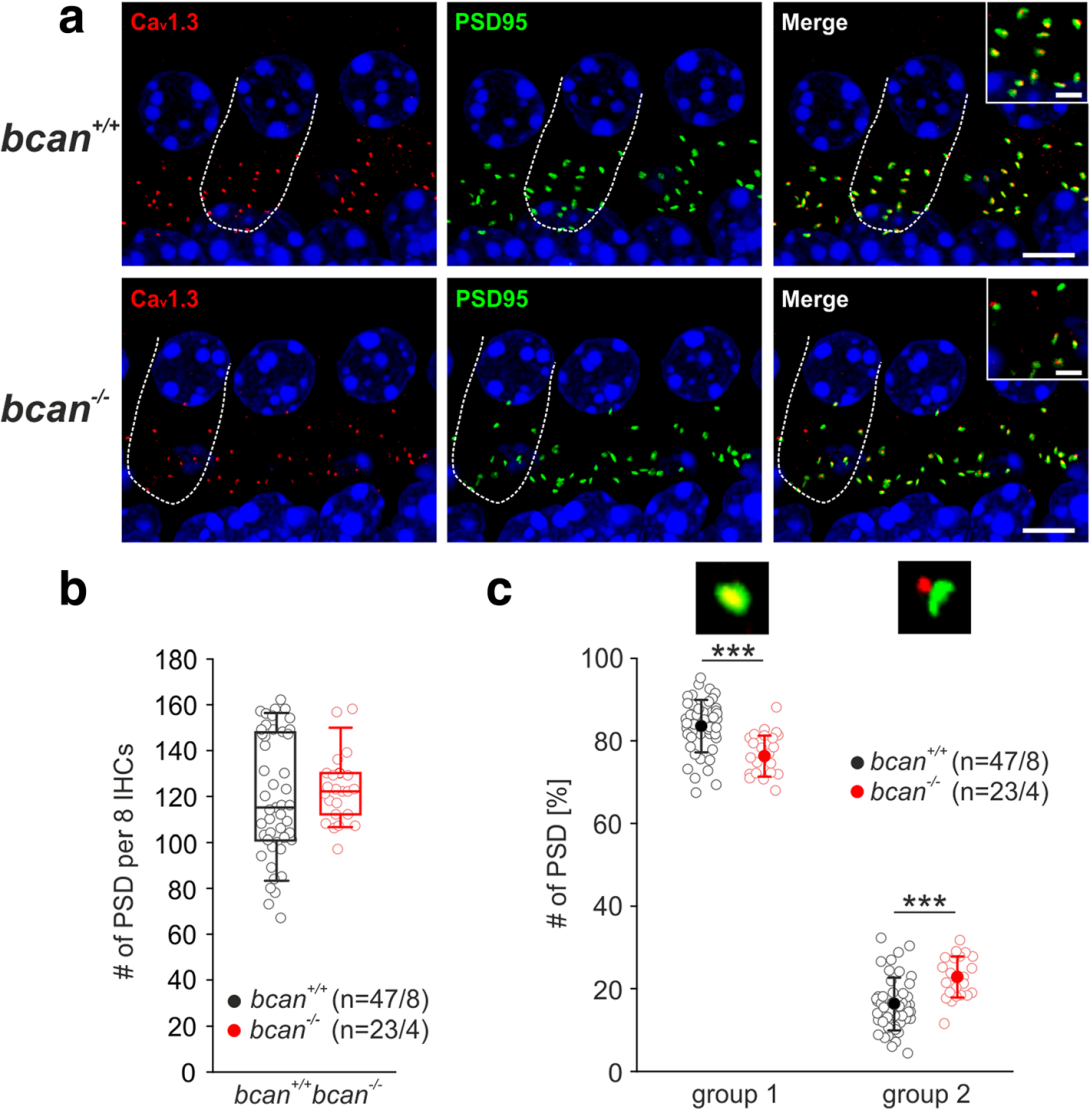
Spatial coupling of presynaptic Ca_v_1.3 and postsynaptic PSD-95 clusters at the IHC synapse in *bcan*^*−/−*^ mice. **a** Immunohistochemical double-labeling of presynaptic Ca_v_1.3 channels (red) and postsynaptic densities (PSD-95, green) in cochlear whole-mount preparations of *bcan*^*+/+*^ and *bcan*^*−/−*^ mice, with one representative IHC indicated by the dashed line. The magnification of the basal part of the labeled IHC is illustrated in the inset in the merge figure. **b** Quantification of the number of postsynaptic densities labeled with anti-PSD-95 did not yield any genotype-specific difference (*bcan*^+/+^ (black): 115 [101,148] per 8 IHCs, *n* = 47 stretches of 8 IHCs of 8 whole-mounts, *bcan*^−/−^ (red): 122 [112,130] per 8 IHCs, *n* = 23 stretches of 8 IHCs of 4 whole-mounts, *p* = 0.409, Mann-Whitney rank sum test). **c** The analysis of the spatial coupling between Ca_v_1.3 channel clusters (red immunosignal) and PSD-95-positive postsynaptic densities (green immunosignal) revealed a significant reduction in co-localized red and green immunopositive punctae (group 1, *p* < 0.001, *t* test) and a significant increase in spatially shifted green and red immunopositive punctae (group 2, *p* < 0.001, *t* test) in *bcan*^*−/−*^ mice. The immunopositive spots were quantified in maximum intensity projections of confocal stacks of stretches of 8 IHCs (*bcan*^*+/+*^: *n* = 47; *bcan*^*−/−*^: *n* = 23) imaged from 8 *bcan*^*+/+*^ whole-mount preparations and 4 *bcan*^*−/−*^ whole-mount preparations. Scales 5 μm, insets 2.5 μm, *** *p* < 0.001. Data are presented as median [first quartile, third quartile] in **b** and as mean ± S.D. in **c**

The reduced hearing sensitivity and weakly increased response latencies of *bcan*^*−/−*^ mice might point to impairment of synaptic transmission at the IHC synapse. A characteristic feature of the afferent ribbon synapse is its ability to faithfully transmit signals with high temporal precision and at high rates which requires—among other factors—tight, spatial alignment of presynaptic release sites and postsynaptic receptors. In a recent report by Fell and colleagues [32], the ECM was suggested to play a key role in the spatial coupling of pre- and postsynaptic elements by interacting with presynaptic α_2_δ2/Ca_v_1.3 channels and postsynaptic structures. We hypothesize that brevican contributes to spatial coupling between pre- and postsynaptic elements as previous results demonstrated a physical interaction of brevican with the AMPA receptor subunits GluR1, GluR2/3, and GluR4 [33, 34]. We tested if the genetic deletion of brevican has any direct effect on the spatial coupling of presynaptic Ca_v_1.3 channels and the postsynaptic scaffold protein PSD-95. Ca_v_1.3 channels and PSD-95 were immunohistochemically labeled in apical and midbasal turns of cochleae of *bcan*^*+/+*^ and *bcan*^*−/−*^ mice, and high-resolution z-stack confocal images were acquired (Fig. 9a). The analysis was based on the quantification of the number of PSD-95 immuno-positive punctae that were either completely co-localized (group 1, Fig. 9c) or not co-localized (group 2, Fig. 9c) with Ca_v_1.3-positive punctae. The total number of PSD-95 immuno-positive punctae did not vary between wildtype and brevican-deficient mice (Fig. 9b; *bcan*^+/+^: 115 [101,148] per 8 IHCs, *n* = 47 stretches of 8 IHCs of 8 whole-mounts, *bcan*^−/−^: 122 [112,130] per 8 IHCs, *n* = 23 stretches of 8 IHCs of 4 whole-mounts, *p* = 0.409, Mann-Whitney rank sum test). Still, the relative number of PSD-95-labeled spots that were colocalized with Ca_v_1.3-labeled spots (group 1) was significantly reduced in *bcan*^*−/−*^ mice (*bcan*^+/+^: 83.5 ± 6.3%, *n* = 47 stretches of 8 IHCs of 8 whole-mounts, *bcan*^−/−^: 77.1 ± 5%, *n* = 23 stretches of 8 IHCs of 4 whole-mounts, *p* < 0.001, *t* test). On the contrary, the relative number of dislocated PSD-95-labeled and Ca_v_1.3-labeled spots was significantly increased (*bcan*^+/+^: 16.5 ± 6.3%, *n* = 47 stretches of 8 IHCs of 8 whole-mounts, *bcan*^−/−^: 22.9 ± 5%, *n* = 23 stretches of 8 IHCs of 4 whole-mounts, *p* < 0.001, *t* test, Fig. 9b).

In conclusion, these data show that the close apposition of pre- and postsynaptic elements was significantly reduced in the absence of brevican, indicating that this CSPG contributes to the tight, spatial coupling of presynaptic release sites and postsynaptic receptors, which is an important precondition for ultrafast and temporally precise synaptic transmission at the hair cell ribbon synapse.

## Discussion

The present data demonstrate that IHCs in the cochlea are embedded in a basket-like agglomeration of ECM molecules, including the CSPGs brevican, aggrecan, and neurocan as well as the HAPLN 1, HAPLN4, and tenascin-R. In this ECM basket covering, the basal part of the IHC where synaptic contacts are formed, brevican was the major proteoglycan. Genetic deletion of brevican was linked to mildly impaired hearing function which was connected to an increased dislocation of clusters of presynaptic Ca_v_1.3 channels and postsynaptic density protein PSD-95. These data suggest a significant role of brevican in the spatial alignment of presynaptic release sites and postsynaptic densities, which is an essential precondition for precise and ultrafast synaptic transmission at the IHC synapse.

### Distribution of aggrecan and brevican in the cochlea

The CSPGs aggrecan and brevican are two main components of PNNs of neurons in the CNS [35]. In the present work, we demonstrate that these CSPGs are also expressed at sensory IHCs in the auditory periphery and beyond that in other regions of the organ of Corti, which at least in the case of brevican was a surprising finding.

Brevican appeared as a prominent, thin layer between the otic capsule and the spiral ligament. The brevican immunolabeling matched the localization of the layer of type III fibrocytes that form the border to the otic capsule [36] and mediate the anchoring of the spiral ligament to the bony wall [37]. Brevican was further found to be expressed in the osseous spiral lamina that is situated between the spiral limbus and the auditory nerve fibers. These results were not expected at all since brevican was assumed to be a nervous tissue specific molecule, typically characterized by a perisynaptic location, e.g., in PNNs and axonal coats, or being expressed at the nodes of Ranvier [38]. On the one hand, the present work demonstrated that brevican is not only CNS-specific but is also expressed—at least in the auditory—peripheral nerve tissue. Though, at IHCs brevican was strongly expressed in the perisynaptic space, confirming findings from the CNS, brevican was obviously not always associated with synapses, as indicated by the presently described thin layer of brevican between bony tissues and attached, connective tissue in the cochlea. The function of brevican between those tissues is unknown. It might be a constituent of basement membranes that are found between connective tissues and epithelial cells in the cochlea and were reported to contain proteoglycans, but so far only heparan-sulfated proteoglycans were detected in those membranes [24].

Aggrecan was found to be expressed in the spiral limbus and in parts of the spiral ligament that contact the lateral wall and correspond to the region of type IV fibrocytes [36]. These findings complement and specify previous results obtained in the chinchilla and rat cochlea which reported the presence of unspecified chondroitin-sulfated and cartilage-specific heparan-sulfated proteoglycans in the spiral limbus and spiral ligament [22, 23]. Ultrastructural analyses revealed that the composition of the spiral limbus is comparable to a premature state of cartilage [22], which might explain the strong expression of the cartilage-specific proteoglycan aggrecan within this structure. In contrast to the strong aggrecan labeling in the spiral limbus and spiral ligament, only little aggrecan was found at the IHCs suggesting that aggrecan might be of minor relevance for the structure and function of the ECM complex surrounding hair cells. Though the specific function of aggrecan in the spiral limbus and ligament is unknown, it may well be that due to its strong anionic charge aggrecan contributes to spatial ion buffering and recycling [39, 40] and to maintenance of the ion compositions of perilymph and endolymph. In addition, aggrecan might promote resistance against compression, an aggrecan-specific function in cartilage [41], and thus might contribute to the stability of the organ of Corti.

### ECM basket at IHCs vs. PNNs of neurons in the CNS—similarities and differences

PNNs are specialized ECM structures that enclose somata and proximal dendrites of specific types of neurons in the CNS, thereby tightly wrapping around synapses [2, 42–46]. Similarly, the cochlear hair cell ECM was found to be strongly associated with synapses as it exclusively covered the basal part of IHCs coinciding with the appearance of pre- and postsynaptic structures. In addition, the IHC ECM baskets were restricted to the unmyelinated part of innervating fibers (data not shown), which is also valid for PNNs of neurons [46, 47]. Besides these common features, we also found substantial differences in the molecular fine structure of IHC ECM basket and PNNs. While aggrecan is a prominent constituent of PNNs in the CNS [35, 39, 48–50], this proteoglycan (as well as neurocan) seems to be only sparsely present within the ECM complex surrounding IHCs. Instead, the structure of the hair cell ECM basket is based on brevican, which interacts with HAPLN1 and HAPLN4. Though it cannot be excluded that additional ECM molecules contribute to the hair cell ECM (e.g., versican), the massive degradation of the IHC ECM basket in brevican-deficient mice suggests that brevican serves as the primary organizer of the structure and potentially also the function of those ECM baskets.

In addition to the common aggrecan-based, somatodendritic PNNs, a second type of CSPG-based matrix, termed axonal coats, has been recently identified in the brain [51–54]. These oval or round-shaped ECM aggregates, which enclose individual synapses primarily terminating on dendrites, were shown to be mainly based on the proteoglycan brevican [51, 52, 55]. The function of the axonal coats remains elusive, but it is speculated that these matrix assemblies stabilize the synapse-dendrite interaction [53]. From a morphological point of view, the IHCs and peripheral dendrites of spiral ganglion neurons that contact the IHCs also reflect synapse-dendrite junctions. Thus, the ECM baskets at IHCs might be the peripheral equivalent to axonal coats in the brain, at least when considering the composition of the ECM baskets at IHCs. Still, the structure and expansion of the ECM at IHCs is much more complex than axonal coats which typically surround only individual synapses. In contrast to the situation at the IHC, our observations at OHCs, where brevican and HAPLN1 were occasionally found to enclose individual ribbon synapses fully match the descriptions of axonal coats on neuronal dendrites. Taken together, the ECM basket at IHCs might be classified as a new, intermediate type of pericellular ECM, representing a structural mix of PNNs and axonal coats.

Surprisingly, the cell-specific ECM at IHCs has merely been examined. The little data that is available demonstrates a glycoprotein coat at the apical (endolymphatic) surface of IHCs that carries the stereovilli [25]. There is to our knowledge no information about the specific molecules forming this apical ECM coat. In the present study, we never observed any of the tested ECM molecules at the apical pole of the IHCs. We therefore conclude that this earlier reported glycoprotein coat is neither made up of brevican, aggrecan, neurocan, tenascin-R, nor HAPLN1/4.

### Contribution of brevican to the spatial coupling of pre- and postsynaptic structures and its impact on hearing function

Brevican is typically expressed in close vicinity to synapses [19, 38, 46] and is assumed to interact with and stabilize synaptic molecules, e.g., by controlling lateral diffusion of AMPA receptors [46, 56].

The results of the present work even expand this idea by demonstrating that brevican not only interacts with individual synaptic molecules but also contributes to the transsynaptic alignment of corresponding pre- and postsynaptic elements. In the absence of brevican, the tight spatial coupling of presynaptic Ca_v_1.3 channels and postsynaptic density proteins PSD-95 was partially impaired at IHC synapses. This function is likely to be based on the direct physical interaction between brevican and synaptic proteins which is supported by recent results obtained in hippocampal cells where brevican was shown to directly bind to glutamate receptor subunits GluR1, GluR2/3, and GluR4 [33, 34].

Interestingly, a functional null mutant mouse line deficient for the α_2_δ2 subunit of the presynaptic Ca_v_1.3 channel showed a similarly reduced spatial alignment of Ca_v_1.3 channel and PSD-95 clusters at the IHC synapse [32]. The authors of this study hypothesized that the α_2_δ2 subunit might bind to unknown extracellular matrix proteins and thereby conjointly control the spatial coupling of presynaptic calcium channels and postsynaptic glutamate receptors. Our data strongly support this hypothesis and implicate brevican as a key molecule for this. It remains to be investigated whether brevican interacts with α_2_δ2 or whether other extracellular proteins are involved, e.g., adhesion molecules such as neuroligins and neurexins [57]. Further, we cannot exclude that other ECM components contribute to the coordination of pre- and postsynaptic apposition, e.g., tenascin-R, HAPLN1, and HAPLN4 as well as neurocan and aggrecan, since these molecules were also absent or greatly reduced at IHCs in brevican-deficient mice.

The complex ECM basket structure at the IHC may serve yet another function, the chemical isolation of its individual synapses. Each of the 12–17 presynaptic ribbons of one IHC drives the peripheral dendrite of one spiral ganglion neuron, which can be classified into three fiber types with different spontaneous rates and dynamic ranges (for review see [58]). A differentiation among presynaptic ribbons [58, 59] as well as postsynaptic neurons [60] is likely necessary to enable an IHC to code for 80-dB level information. Physically separating the individual synapses around the basolateral pole of an IHC by small brevican-based baskets would prevent spillover of glutamate to “wrong” synapses and its dilution and therefore better preserve level information as well as timing. Together with the tight spatial coupling of the presynaptic release sites and postsynaptic glutamate receptors, these effects could contribute to a very sensitive, temporally precise and ultrafast transmission within a large dynamic range, all of which are required for proper processing of auditory information.

At a central auditory synapse, the calyx of Held in the MNTB, the absence of brevican resulted in delayed postsynaptic responses due to a reduction of speed of synaptic transmission [17]. The mechanisms behind are still unknown. Though electron microscopical analyses revealed significant changes in the ultrastructure of the calyx of Held, indicating a disruption in the compartmentalization of the subsynaptic space [17, 46], it might be interesting to scrutinize whether the transsynaptic alignment of active zones and postsynaptic densities are also affected at the calyx of Held synapse in brevican-deficient mice and even at other synapses in the CNS.

## Conclusions

In conclusion, the present data provide to our knowledge the first description of pericellular, brevican-based ECM complexes in the auditory periphery which enclose synapses contacting the IHCs at their basal pole and might be functionally related to PNNs of neurons in the CNS.

## Methods

### Animals

Experiments were conducted in transgenic mice deficient for the CSPG brevican (background: C57BL/6N). Both homozygous knockout mice (*bcan*^−/−^) and wildtype littermates (*bcan*^+/+^) derived from heterozygous parents. The animals were housed in the animal care facilities of the Experimental center of the Faculty of Medicine of the University of Leipzig and of Saarland University in a temperature-controlled environment with free access to food and water and 12-h dark/light cycle. The genotype of the experimental animals was determined by PCR. Mice of both sexes were used.

### Immunohistochemistry in cochlear whole-mounts

Mice (P26-P30, *n* = 15/10 *bcan*^+/+^/*bcan*^−/−^) were deeply anesthetized with CO_2_, and cochleae (*n* = 30/20 *bcan*^+/+^/*bcan*^−/−^) were isolated and fixed by intracochlear perfusion through the round and oval window with either 2% paraformaldehyde (PFA) or Zamboni’s fixative for 10 min [61]. After rinsing with PBS, the cochleae were dissected in apical, middle, and basal turns (two to eight whole-mounts were acquired per animal). The whole-mounts were transferred to SuperFrost Plus slides (Fisher Scientific) and attached to the surface using Cell-Tak (Corning). Before immunolabeling, whole-mounts were treated with permeabilization solution (0.5% Triton X-100 in PBS) for 10 min and blocking solution (1% bovine serum albumin in PBS) for 30 min. Primary antibodies were incubated in blocking solution for 24 h at 4 °C. The whole-mounts were stained with antibodies against brevican (rabbit polyclonal, 1:2000, B756, gift of R.T. Matthews and mouse monoclonal, 1:1000, BD Biosciences, RRID:AB_398212), aggrecan (rabbit polyclonal, 1:1000, AB1031, Millipore, RRID:AB_90460), neurocan (sheep polyclonal, 1:400, R&D systems, RRID:AB_2044705), HAPLN1 (goat polyclonal, 1:100, R&D Systems, RRID:AB_2116134), HAPLN4 (goat polyclonal, 1:100, R&D Systems, RRID:AB_2116264), tenascin-R (mouse monoclonal, 1:1000, Synaptic Systems, RRID:AB2256347), CtBP2 (mouse monoclonal, 1:100, BD Biosciences, RRID:AB_399431), myelin basic protein (MBP) (rat monoclonal, 1:400, Abcam, RRID:AB_305869), neurofilament (mouse monoclonal, 1:1000, SMI32, Sternberger Monoclonals, RRID:AB_2315331), GluR2/3 (rabbit polyclonal, 1:1000, Millipore, RRID:AB_11212089), GluR4 (rabbit monoclonal, 1:1000, Cell-Signaling, RRID:AB_10829469), Ca_v_1.3 (rabbit polyclonal, 1:500, Alomone labs, RRID:AB_2039775), PSD-95 (mouse monoclonal, 1:1000, NeuroMab, RRID:AB_2315909), and vGlut3 (guinea pig polyclonal, Synaptic Systems, 1:250, RRID:AB_2619825). Primary antibodies were detected by fluorescent secondary antibodies (Cy2- or Cy3-conjugated, 1:1000; Cy5 conjugated, 1:800, DIANOVA; Alexa488-conjugated 1:1000, Invitrogen), and nuclei were stained using DAPI (1:2000, Sigma-Aldrich). The applied antibodies were either well characterized elsewhere (anti-aggrecan AB1031: [47]; anti-HAPLN4: [62]; anti-HAPLN1: [1]; anti-brevican: [27]; anti-neurocan: [63]; anti-vGlut3: [21]; Ca_v_1.3: [32]; anti-CtBP2: [64]; anti-GluR2/3: [65]; anti-PSD-95: [32]; anti-GluR4: [66]; anti-MBP: [67]; anti-SMI32: [68]) or specificity was confirmed by using specific knockout lines (*bcan*^*−/−*^*, acan*^*+/−*^*, ncan*^*−/−*^*, hapln1*^*−/−*^*, tenR*^*−/−*^).

### Immunohistochemistry of frozen cochlea sections

Following CO_2_ anesthesia, cochleae (*n* = 3) were dissected from P27–P30 wildtype mice (*n* = 3), perfused through the round and oval window with 4% PFA, and kept in the fixative for 60 min [69]. Following rinsing with PBS, cochleae were decalcified with rapid bone decalcifier for 15 min and rinsed again with PBS. For frozen sectioning, the cochleae were cryoprotected in 10% sucrose for 30 min and in 15% sucrose overnight. After embedding in O.C.T compound (Tissue-Tek®), the cochlea tissue was cut into sections of 12–16 μm on a cryostat (HM 500 OM, Microm), collected on SuperFrost Plus Slides (Fisher Scientific), and stored at − 20 °C. Prior to immunostaining, frozen sections were thawed, postfixed again in 2% PFA for 5 min and washed with PBS. Then, the sections were permeabilized with 0.5% Triton X-100 in PBS for 10 min and incubated in blocking solution (1% bovine serum albumin in PBS) for 30 min. Primary antibodies were incubated in blocking solution for 24 h at 4 °C. Antibodies against brevican (1:2000, B756, gift of R.T. Matthews and 1:1000, BD Biosciences, RRID:AB_398212), aggrecan (1:1000, AB1031, Millipore, RRID:AB_90460), neurocan (1:400, R&D systems, RRID:AB_2044705), HAPLN1 (1:100, R&D Systems, RRID:AB_2116134), and calbindin (rabbit polyclonal, 1:1500, CB38, SWANT, RRID:AB_2721225) were used and detected by fluorescent secondary antibodies (Cy2 or Cy3 conjugated, 1:1000; Cy5 conjugated, 1:800, DIANOVA). Nuclei were stained with DAPI (1:2000, Sigma-Aldrich).

### Hematoxylin-eosin staining of paraffin cochlea sections

Following CO_2_ anesthesia, cochleae (*n* = 3 per genotype) were dissected from P27–P30 mice (*n* = 3 per genotype). For fixation and decalcification the same protocol as used for frozen cochleae was applied. Afterwards, cochleae were embedded in paraffin, cut into 7–10 μm thick sections (SM 2000 R, Leica), and mounted on SuperFrost Plus slides (Fisher Scientific). Paraffin sections were dewaxed with xylol and subsequently stained with hematoxylin-eosin.

### Image acquisition and analysis

Bright-field microscopical images of hematoxylin-eosin-stained paraffin sections were acquired with a Keyence microscope (BZ-9000, Keyence, Neu-Isenburg, Germany). Whole-mounts and cryosections were analyzed with a Zeiss confocal laser scanning microscope (LSM 510 Meta and LSM 880 with Airyscan, Zeiss Microscopy GmbH, Jena, Germany). Images were obtained using a 63x water immersion objective (C-Apochromat, Zeiss) with 1.2 numerical aperture. The confocal pinhole was set to 1 airy unit. Stacks along the *z*-axis were taken at distances of 1 μm. Maximum intensity projections (MIPs) were generated and analyzed using ZEN 2.3 (Zeiss Microscopy GmbH, Jena, Germany) and Photoshop CS5 (Adobe System, Mountain View, CA, USA).

For quantification of the number of ribbon synapses, five apical whole-mounts were acquired from five *bcan*^+/+^ and four *bcan*^−/−^ mice and five midbasal whole-mounts were acquired from three *bcan*^+/+^ and three *bcan*^−/−^ mice. CtBP2-positive punctae were quantified at 6–10 IHCs/whole-mount and the number of CtBP2-positive punctae per IHC was determined for each whole-mount.

For quantification of the overlap of Ca_v_1.3 and PSD-95, z-stacks (optical slice thickness: 0.32 μm) of whole-mounts double-labeled with antibodies against Ca_v_1.3 and PSD-95 were acquired with a Zeiss confocal laser scanning microscope (LSM 710, Zeiss Microcopy GmbH, Göttingen, Germany) using a 63x oil objective (1.4 numerical aperture, Planapochromat, Zeiss), thereby covering the complete basolateral pole of the IHC. After calculation of maximum intensity projections, the background was subtracted and contrast and brightness were set to the same level for all images, each containing eight adjacent IHCs (total length, 67.48 μm). The software ZEN 2.3 (Zeiss, Jena, Germany) was used to identify the immuno-positive PSD-95 spots in each image which were classified as either being co-localized with a Ca_v_1.3 immuno-positive spot (group 1) or as not being co-localized with a Ca_v_1.3 immuno-positive spot (group 2). Only spots with a minimum size of 45,000 nm^2^ were included into the dataset. This analysis was performed on 47 stretches of IHCs in 8/2 *bcan*^+/+^ whole-mounts/animals and 23 stretches of 8 IHCs in 4/1 *bcan*^−/−^ whole-mounts/animals.

### Western blot

Western blotting was performed to confirm the presence of the immunohistochemically detected PNN subcomponents. Cochleae (*n* = 30) of 15 C57BL/6N mice (P35) were isolated and immediately shock frozen in liquid nitrogen. Cochlear tissue was homogenized with 300 μl of homogenizing buffer (Tissue Extraction Reagent I, ThermoFischer Scientific) and complete protease inhibitor (Roche) using Precellys® 24 (VWR). The homogenate was centrifuged at 10,000*g* for 15 min at 4 °C, and the supernatant was decanted. For analysis of the core protein of aggrecan, 100 μl of the supernatant (~ 700 μg total protein) was digested with 0.05 units chondroitinase (Sigma-Aldrich, according to [49]) in Tris-HCL (pH 8.0) for 3.5 h at 37 °C. The supernatants were further denatured with laemmli sample buffer at 95 °C for 5 min. Proteins were electrophoretically separated in a 6% SDS-PA gel (brevican, aggrecan, neurocan, HAPLN1) or 8% SDS-PA gel (HAPLN4) and electro-transferred onto a poly vinylidene difluoride membrane (PVDF, Roche) over night at 20 V. After blocking with 1% BSA in TBS-T (0.05% Tween-20), the PVDF membrane was incubated with the primary antibodies overnight at 4 °C (brevican: 1:1250, BD Biosciences, RRID:AB_398212; aggrecan: 1:1000, AB1031, Millipore, RRID:AB_90460; neurocan: 1:4000, R&D systems, RRID:AB_2044705; HAPLN1: 1:1000, R&D Systems, RRID:AB_2116134; HAPLN4: 1:1000, R&D Systems, RRID:AB_2116264). The blots were washed in TBS-T and incubated for 1 h at room temperature with 1:10000 diluted peroxidase-conjugated secondary antibody. The protein bands were detected with an enhanced chemoluminescence detection system (DNR Bio-Imaging system, Biostep, Germany).

### Auditory brainstem responses and distortion product otoacoustic emissions

Hearing performance was tested by ABR and DPOAE measurements in 5-week-old mice (ABR: *n* = 6/12 animals/ears each genotype; DPOAE: *bcan*^+/+^, *n* = 7/13 animals/ears, *bcan*^−/−^, *n* = 6/12 animals/ears) as previously described [32]. Mice were anesthetized, using a mixture of ketamine-hydrochloride (75 mg/kg body weight, Ketavet 100, Pharmacia) and xylazine hydrochloride (5 mg/kg body weight, Rompun 290, Bayer), which was initially injected intraperitoneally. Anesthesia was maintained by subcutaneous injections of one third of the initial dose approximately every 30 min. Body temperature was kept at 37–38 °C by placing the mice on a temperature-controlled heating pad.

For ABR recordings, electrodes were placed at the ear (positive) and vertex (negative). ABR thresholds were determined using either click stimuli (100 μs, 512 repetitions) or pure tone stimuli (3 ms, 1 ms rise and fall time, 256 repetitions) within a frequency range of 2–45 kHz. Latencies of ABR waves I to IV were determined for levels of 15 to 60 dB above threshold.

DPOAE amplitudes were measured using the cubic 2f1-f2 paradigm, where f1 and f2 are primary pure tones with f2 = 1.22 × f1. The sound pressure level was L1 = 55 dB SPL for the first primary tone and L2 = 45 dB SPL for the second primary tone. DPOAE amplitudes were determined in the range of 10–18 kHz (0.5 kHz step size) and averaged [70, 71].

### Whole-cell patch clamp recordings in IHCs

For recording Ba^2+^ currents through calcium channels [32], cochleae of P20–P21 mice (*n* = 2 *bcan*^+/+^ mice and *n* = 2 *bcan*^−/−^ mice) were acutely dissected and superfused with bath solution containing (in mM): 72 lactobionate-NaOH, 40 NaCl, 35 TEA, 15 4-AP, 10 BaCl_2_, 10 HEPES, 5.6 KCl, 5.3 glucose, and 1 MgCl_2_ (pH 7.35, osmolarity 320 mosmol/kg). The pipette solution contained the following (in mM): 110 Cs^+^-methane sulfonate, 20 CsCl, 10 Na^+^ phosphocreatine, 5 HEPES, 5 EGTA, 4 MgCl_2_, 4 Na_2_ATP, 0.3 GTP, and 0.1 CaCl_2_ (pH 7.35, osmolarity 305 mosmol/kg). Series resistance was corrected by 80–95%, linear leak subtraction was performed off-line, and voltages were corrected by subtracting a liquid junction potential of 8 mV. For each IHC, the peak Ba^2+^ current was determined from the *I-V* curve obtained by averaging the current from 7 to 8 ms as a function of the voltage step. *I-V* curves of Ba^2+^ currents were fitted to a second-order Boltzmann function times Goldman-Hodgkin-Katz driving force to determine parameters of the activation curve, the voltage of half-maximum activation, *V*_*h*_, and the voltage sensitivity of activation, the slope factor *k*, according to [32].

### Statistics

Data are provided as mean ± S.D. or median [first quartile, third quartile]. For comparison between genotypes, two-tailed *t* test was used or Mann-Whitney rank sum test, depending on distribution of the data. ABR growth functions of latencies were not tested with ANOVA because of the unequal variances. Rather, a correlation analysis with calculation of regression lines was performed, and parameters of the regression lines (slopes and *y*-axis intercepts) were tested to differences according to Sachs [72] (two-tailed, *α* = 0.05). A detailed list of number of samples, data analyses, and statistics is provided in Additional file 4.

## Additional files

Additional file 1: **Figure S1.** Immunohistochemical localization of HAPLN1 at IHCs in cross-sections of the mouse cochlea. A, B HAPLN1 labeling (green) yields a strong immunosignal in the temporal bone and at calbindin-positive IHCs (red; B, magnification of white box in A). Maximum intensity projections of confocal stacks of cochlear cross-sections. A, scale 50 μm, B, scale 5 μm. (TIF 3300 kb)

Additional file 2: **Figure S2.** Hearing function assessed by ABR. Individual ABR thresholds in response to pure tones of *n* = 6/12 ears/animals each genotype (*bcan*^*+/+*^, black, left; *bcan*^*−/−*^, red, right). (TIF 3840 kb)

Additional file 3: **Figure S3.** Growth functions of latencies of ABR waves I to IV. Growth functions of the latencies of ABR wave I, II, III, and IV are illustrated for each individual (*n* = 6/12 ears/animals each genotype, *bcan*^*+/+*^, black, upper row; *bcan*^*−/−*^, red, lower row). (TIF 10387 kb)

Additional file 4: A detailed list of number of samples, data analyses, and statistics [72]. (XLSX 13 kb)

## Abbreviations

ABR: Auditory brainstem response
ACAN: Aggrecan
BCAN: Brevican
CNS: Central nervous system
CSPG: Chondroitin-sulfated proteoglycan
DPOAE: Distortion product otoacoustic emission
ECM: Extracellular matrix
HAPLN: Hyaluronan and proteoglycan link protein
IHC: Inner hair cell
MNTB: Medial nucleus of the trapezoid body
NCAN: Neurocan
OHC: Outer hair cell
PNN: Perineuronal net
SL: Spiral limbus
SPL: Sound pressure level
TenR: Tenascin-R
