# Supplementary figures and images for: Synaptic coupling of inner ear sensory cells is controlled by brevican-based extracellular matrix baskets resembling perineuronal nets
Source: BMC Biol. 2018 Sep 26;16:99. doi: 10.1186/s12915-018-0566-8 (PMC6156866; doi:10.1186/s12915-018-0566-8)

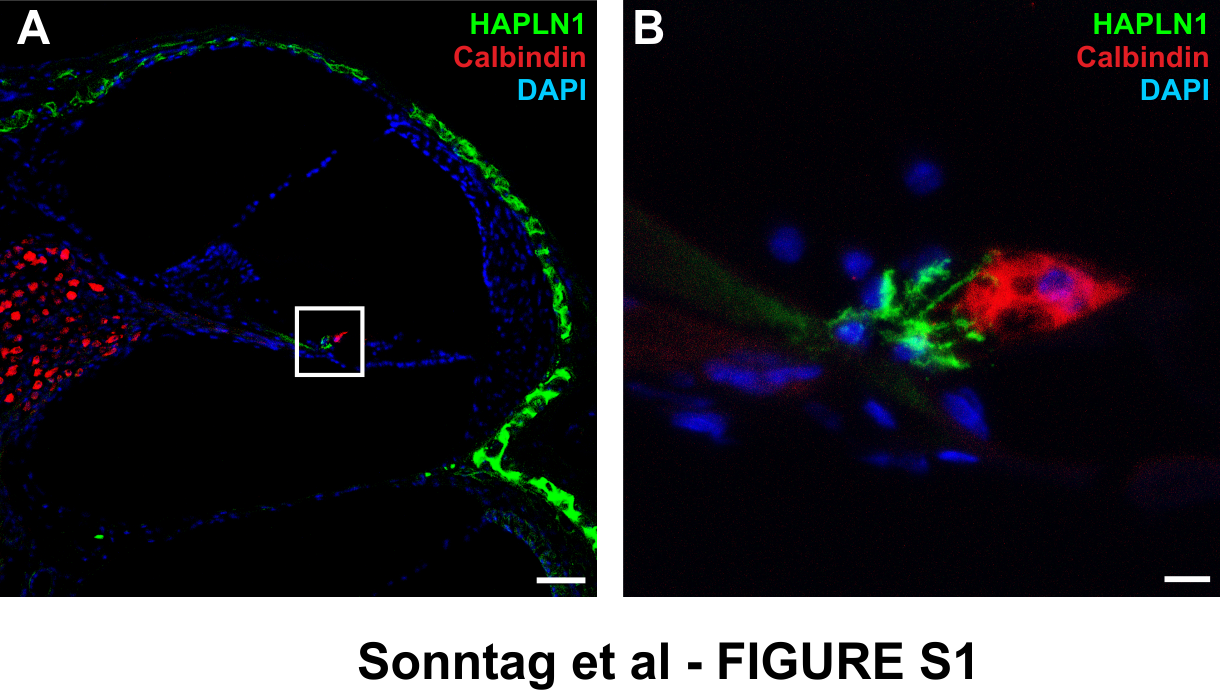

Supplement: Supplementary file 1 — Figure S1. Immunohistochemical localization of HAPLN1 at IHCs in cross-sections of the mouse cochlea. A, B HAPLN1 labeling (green) yields a strong immunosignal in the temporal bone and at calbindin-positive IHCs (red; B, magnification of white box in A). Maximum intensity projections of confocal stacks of cochlear cross-sections. A, scale 50 μm, B, scale 5 μm. (TIF 3300 kb) [file 12915_2018_566_MOESM1_ESM.tif]

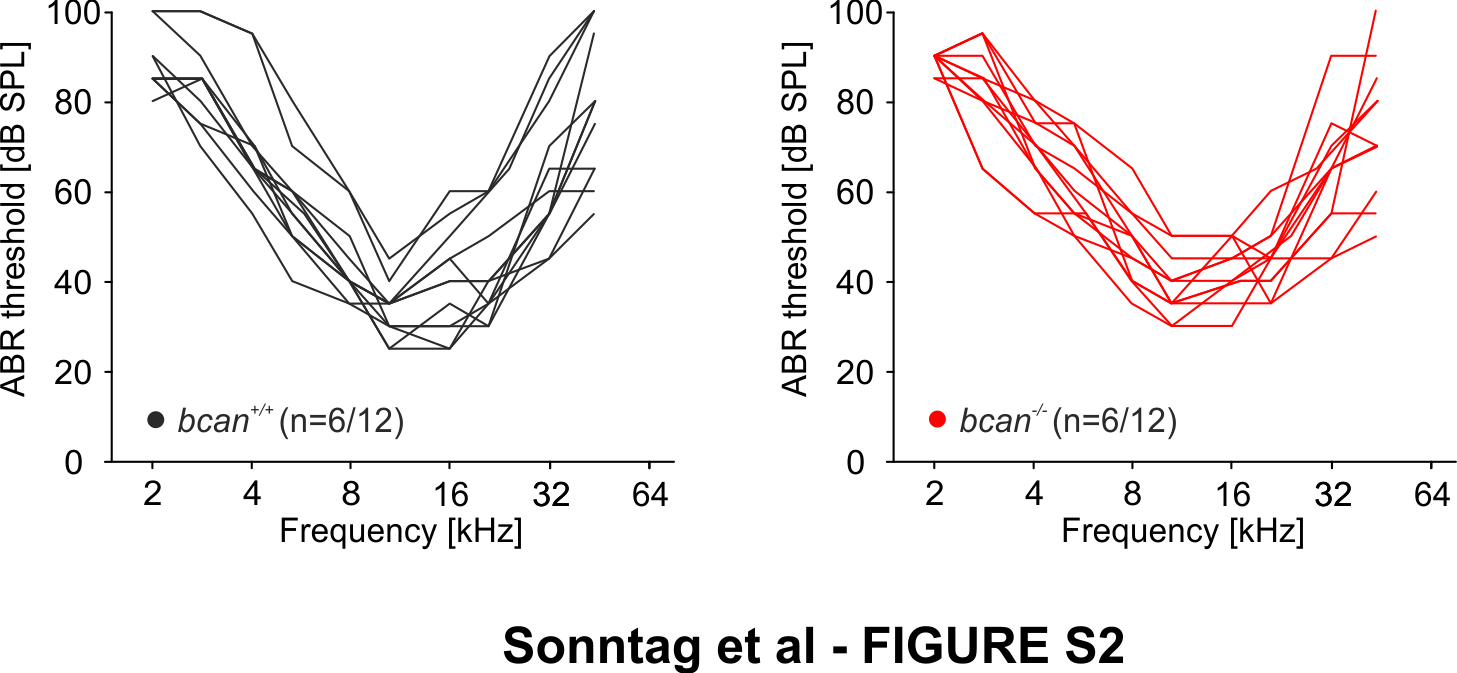

Supplement: Supplementary file 2 — Figure S2. Hearing function assessed by ABR. Individual ABR thresholds in response to pure tones of n = 6/12 ears/animals each genotype (bcan+/+, black, left; bcan−/−, red, right). (TIF 3840 kb) [file 12915_2018_566_MOESM2_ESM.tif]

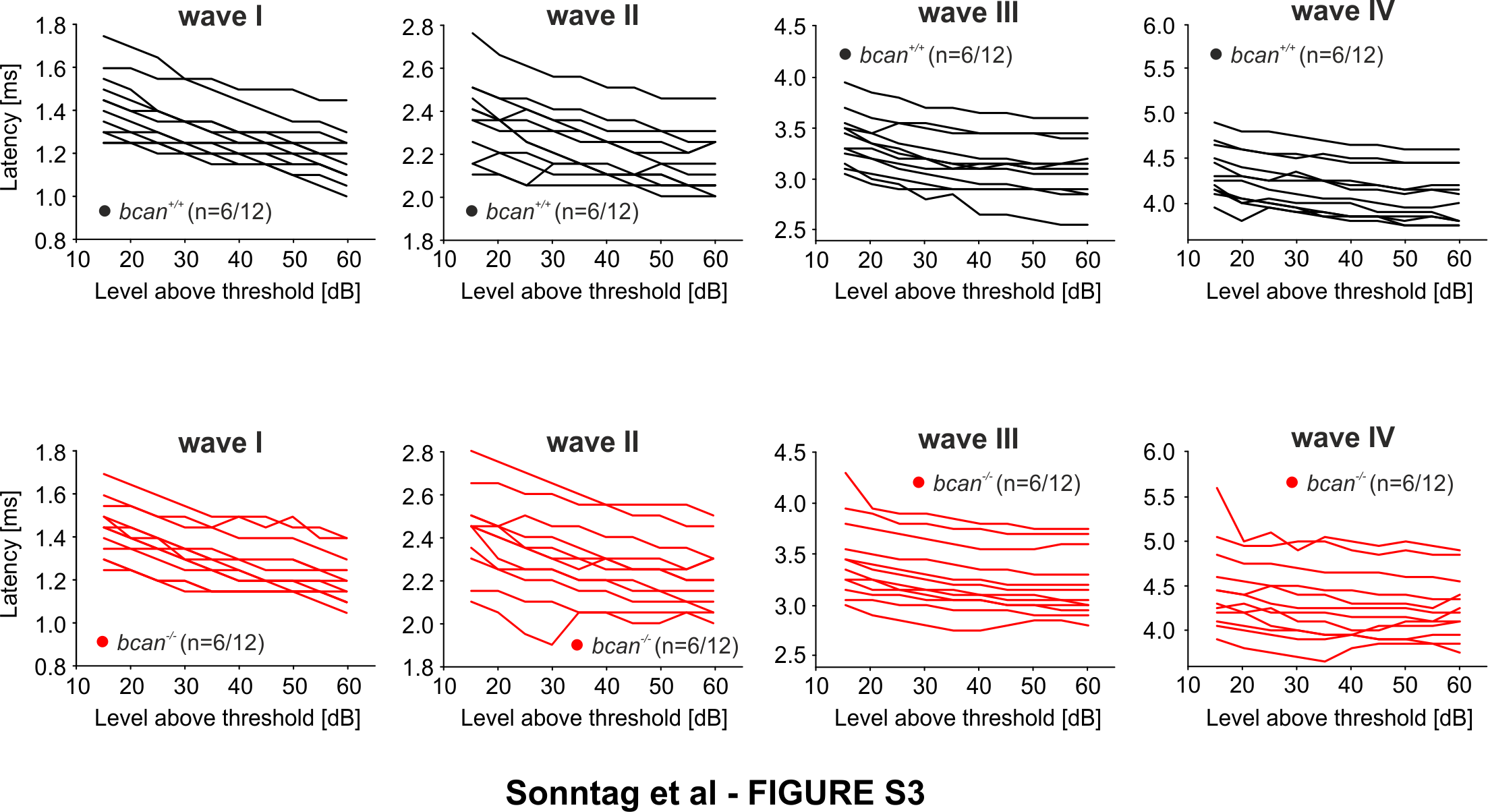

Supplement: Supplementary file 3 — Figure S3. Growth functions of latencies of ABR waves I to IV. Growth functions of the latencies of ABR wave I, II, III, and IV are illustrated for each individual (n = 6/12 ears/animals each genotype, bcan+/+, black, upper row; bcan−/−, red, lower row). (TIF 10387 kb) [file 12915_2018_566_MOESM3_ESM.tif]
